# Nutraceuticals and Functional Foods: Is It Possible and Sustainable for Bridging Health and Food?

**DOI:** 10.3390/foods11111608

**Published:** 2022-05-30

**Authors:** Antonello Santini

**Affiliations:** Department of Pharmacy, University of Napoli Federico II, Via D. Montesano 49, 80131 Napoli, Italy; asantini@unina.it; Tel.: +39-81-2539317

This editorial is part of the Special Issue entitled “Nutraceuticals and Functional Foods: Bridging Health and Food Under a New Perspective”. Current state-of-the-art research on nutraceuticals and functional foods is focusing on the recovery and re-use of bioactive substances from vegetal or animal byproducts. The following aspects of this research must be considered: (i) low environmental impact; (ii) safety of novel extraction strategies; and (iii) new sources (e.g., byproducts from the agro-food area) and new methodologies. Moreover, the byproducts deriving from transformation and manufacturing in the agro-food industry, which are often disposed of or used as feed, must adhere to good manufacturing practices and procedures [1]. Future research in this area includes: (i) the use of non-harmful solvents for the recovery; (ii) zero waste at the end of the processes; (iii) assessing the mechanism of action of nutraceuticals and functional foods. This will present new possibilities for their use as tools of proactive medicine. The expected endpoint is to prevent the onset of health conditions before a pharmacological therapy must be adopted, especially in subjects who do not qualify for a conventional therapeutical approach. This is becoming high desirable, considering the growing demand from the population of safe and all-natural remedies. This may be seen as a sort of “back-to-the-past” approach; however, the original pure holistic approach is presently developing into a more and more scientifically substantiated one. This Special Issue reports research and results of studies addressing some of what is described above. A multidisciplinary and multitarget involvement of different types of expertise is required. The Special Issue ranged from food chemistry to nutrition and from safety to sustainability and new therapeutical approaches, exploiting the role played by nutraceuticals and functional foods.

The health potential of nutraceuticals and functional food is triggering interest in research worldwide on the assessment of their mechanisms of action, which also involves possible interactions with physiological processes, with other molecules or pharmaceuticals, or with foodstuffs themselves. In vitro studies as well as studies on animals and humans must be conducted to exploit and assess these molecules’ targets and mechanisms of action. The aim is to optimize safety, efficacy, and the appropriate formulation for their delivery. This aspect is of utmost importance: once the appropriate organ target is selected, the nutraceuticals and functional food must be allowed to reach it and provide the expected beneficial health effect. Based on this premise, new fields, new applications and emerging areas have been explored in this Special Issue, with an eye open to the predictable future development ahead. For example, there is growing interest in the development of nanonutraceuticals and nanoformulations with better bioavailability, supporting their specific beneficial health effects [2]. The paper by Cicero et al. [3] evaluated mineral and microbiological analyses of spices and aromatic herbs. Spices and aromatic herbs have been documented as a rich source of bioactive compounds that are used for their health benefits and for flavoring and coloring food. Nonetheless, they represent biological hazards and contain chemical substances of concern. The proposed paper gives a snapshot of the research aimed at monitoring the compliance of various spices and aromatic herbs from in the market of a non-European country according to the current European Union and WHO regulations. The results show the safety of the tested spices, which is an important step for their safe use. Lo Vecchio et al. [4] reported the full characterization of *Rhus coriaria* L., a fruit from Sicily which is a potential source of fiber (33.21 ± 1.02%) and unsaturated fatty acids and linoleic and α-linolenic acids (30.82 ± 1.21% and 1.85 ± 0.07%, respectively). Along with the high content in phenolic, anthocyanin, and minerals, its non-toxicity has been confirmed through tests of its extract on zebrafish embryos. This fruit is interesting, since it has also antimicrobial activity, as confirmed in the reported study against multidrug-resistant microorganisms, *Escherichia coli* and *Klebsiella pneumoniae* strains, isolated from poultry. D’Imperio et al. [5] studied the biofortification process of rocket and purslane with Zn. A bioaccessibility study of this nutrient using an in vitro gastrointestinal digestion process confirmed that it is possible to obtain a high content of Zn biofortified rocket and purslane by adopting an appropriate mineral plant nutrition solution enriched with this nutrient. The paper stressed, in fact, the growing interest in the research on these baby leaf vegetables. Biofortification could then be used to reduce the negative impact of mineral malnutrition with a relevant impact on health.

On the same area of interest, the paper by Buturi et al. [6] reported the relevance, novelty and importance of the biofortification of the vegetables with minerals as a tool to improve the human diet. It has been evidenced that the biofortification of vegetables can be a promising strategy to increase the content of specific compounds. The aim is to make them a functional food or source of nutraceuticals in order to formulate new food supplements. The possible interactions occurring at crop level, as well as the bioavailability of different minerals for the consumer, have been taken into consideration in the paper using a wide approach including the quantification of bioavailable fractions.

Santarcangelo et al. [7] reported that the long-ripened Parmigiano Reggiano Italian cheese has a beneficial content of minerals, indicating that it is an interesting food source, of selenium and chromium, among the others, which have beneficial health properties according to European Regulation 432/2012. The characteristics and health-promoting properties of green banana flours have been reported in the study of Khoza et al. [8]. The high content of phenolic and flavonoids and the antioxidant activity evidenced how the flours from Grand Naine and FHIA-01 GBF banana cultivars could potentially be used as raw materials for functional bakery products as well as for the fortification of snacks.

An alternative food source has been described by Mohamad Nasir et al. [9], who focused on edible bird nests, which are consumed as a Chinese traditional food for their health and medicinal purposes due to their high nutritional value. The study proposes a full characterization of edible bird nests, confirming their high nutritional value. This unconventional food matrix has been evaluated, evidencing how the characteristics and the nutrient-extraction methods may influence the availability of bioactive protein and peptides. The study also stressed the potential use of edible bird nests for their beneficial use, taking advantage of both the composition and content of biological active substances and the nutritional properties made available to human consumption.

Benchagra et al. [10] studied the beneficial antioxidant effect of Moroccan pomegranate against oxidative stress processes. The purpose of this study was to characterize the phenolic, flavonoids, and anthocyanin contents of different parts (namely peel and aril) of the *Sefri* variety of pomegranate. The results show that peel extract was richer in these compounds, especially in Punicalagin A and B. As a result, it was remarked that this Moroccan variety of pomegranate has protective effects against the development of metabolic disorder, cancer, atherosclerosis, and cardiovascular disease. Based on these results, the study of *Sefri* pomegranate extracts could open a new frontier in the fields of food preservation and health supplements.

Krobthong et al. proposed a novel approach aimed at developing safe strategies using natural hypolipidemic products and studied the possibility of the use of nano-liposomal Linzghi hydrolysate as novel functional ingredients in the treatment and prevention of obesity [11]. They studied Lingzhi, an edible fungus, as a potential lipid-suppression stimulant. Their results indicated the use of Lingzhi as a functional anti-hyperlipidemic ingredient. Excessive lipid accumulation is a serious health condition, and this result represents a good insight for the exploration of further lipid accumulation in adipocyte cells. The nano-liposomal Linzghi hydrolysate might serve as a novel functional ingredient in the treatment and prevention of obesity, and also indicates the current interest in new sources for nano-formulation of supplements and nutraceuticals.

Another novel and unconventional approach to food supplements was outlined by Gómez-Fernández et al. [12]. In their paper, the authors evaluated the effects on consumers’ acceptability of a milk chocolate reformulation with alternative sugar sweeteners, probiotics, and ω-3 polyunsaturated fatty acids using milk chocolate as a carrier. The impact could be relevant as a potential functional food for the diabetic population. The authors concluded that the complete assessment of the health benefits of reformulated milk chocolates requires further in vitro, in vivo, and clinical studies. The topic is nonetheless triggering interest, since chocolate can be a candidate for the delivery of bioactive compounds. Due to its acceptable taste for the consumer, it may be a good carrier to formulate new nutraceuticals and functional foods.

Mlcek et al.’s paper describes the use of some edible flowers as foods for their beneficial health and nutritional properties [13]. This topic, although not much explored, is presently triggering interest in edible flowers as a possible food source and also for food supplements and nutraceutical development. The authors’ contribution included the peculiarities of some ornamental edible flowers that represent a novel source of nutraceutical substances with valuable biological properties. The nutritional, chemical, and sensory characteristics and the antioxidant efficacy were explored in this paper, opening up a new area of interest towards flowers, which have so far been considered only as ornamental and beautiful to see in a garden or as a decoration on food preparations. Nonetheless, they may also have nutraceutical interest as a novel food.

The beneficial health-promoting properties of Amaranth, a pseudo-cereal crop, were described in the paper by Baraniak et al. [14], who evidenced the dual aspects of this plant that have been known centuries, i.e., its use as a functional food and as a medicine. Amaranth, in fact, has valuable biological properties, being rich in many phytochemicals and having wide pharmacological activity. Indeed, amaranth-based preparations are used in recipes for dietary supplements, functional food, and medicinal products. The authors concluded that the growth in the knowledge regarding this plant could trigger interest in research to promote its use in the development of innovative technologies in foods, nutraceuticals, and cosmetics industries.

In short, and as a final remark, the contributions presented in this Special Issue provide a snapshot of the current and growing interest in the research into the development of novel food supplements and nutraceuticals in view of building more and new knowledge to bridge food, supplements, nutraceuticals, and health in a coordinated and sustainable proactive approach.

## References

[1] Souto E.B., Silva G.F., Dias-Ferreira J., Zielinska A., Ventura F., Durazzo A., Lucarini M., Novellino E., Santini A. (2020). Nanopharmaceutics: Part I—Clinical Trials Legislation and Good Manufacturing Practices (GMP) of Nanotherapeutics in the EU. Pharmaceutics.

[2] Durazzo A., Nazhand A., Lucarini M., Atanasov A.G., Souto E.B., Novellino E., Capasso R., Santini A. (2020). An Updated Overview on Nanonutraceuticals: Focus on Nanoprebiotics and Nanoprobiotics. Int. J. Mol. Sci..

[3] Cicero N., Gervasi T., Durazzo A., Lucarini M., Macrì A., Nava V., Giarratana F., Tardugno R., Vadalà R., Santini A. (2022). Mineral and Microbiological Analysis of Spices and Aromatic Herbs. Foods.

[4] Lo Vecchio G., Cicero N., Nava V., Macrì A., Gervasi C., Capparucci F., Sciortino M., Avellone G., Benameur Q., Santini A. (2022). Chemical Characterization, Antibacterial Activity, and Embryo Acute Toxicity of Rhus coriaria L. Genotype from Sicily (Italy). Foods.

[5] D’Imperio M., Montesano F.F., Serio F., Santovito E., Parente A. (2022). Mineral Composition and Bioaccessibility in Rocket and Purslane after Zn Biofortification Process. Foods.

[6] Buturi C.V., Mauro R.P., Fogliano V., Leonardi C., Giuffrida F. (2021). Mineral Biofortification of Vegetables as a Tool to Improve Human Diet. Foods.

[7] Santarcangelo C., Baldi A., Ciampaglia R., Dacrema M., Di Minno A., Pizzamiglio V., Tenore G.C., Daglia M. (2022). Long-Aged Parmigiano Reggiano PDO: Trace Element Determination Targeted to Health. Foods.

[8] Khoza M., Kayitesi E., Dlamini B.C. (2021). Physicochemical Characteristics, Microstructure and Health Promoting Properties of Green Banana Flour. Foods.

[9] Mohamad Nasir N.N., Mohamad Ibrahim R., Abu Bakar M.Z., Mahmud R., Ab Razak N.A. (2021). Characterization and Extraction Influence Protein Profiling of Edible Bird’s Nest. Foods.

[10] Benchagra L., Berrougui H., Islam M.O., Ramchoun M., Boulbaroud S., Hajjaji A., Fulop T., Ferretti G., Khalil A. (2021). Antioxidant Effect of Moroccan Pomegranate (*Punica granatum* L. Sefri Variety) Extracts Rich in Punicalagin against the Oxidative Stress Process. Foods.

[11] Krobthong S., Yingchutrakul Y., Visessanguan W., Mahatnirunkul T., Samutrtai P., Chaichana C., Papan P., Choowongkomon K. (2021). Study of the Lipolysis Effect of Nanoliposome-Encapsulated Ganoderma lucidum Protein Hydrolysates on Adipocyte Cells Using Proteomics Approach. Foods.

[12] Gómez-Fernández A.R., Faccinetto-Beltrán P., Orozco-Sánchez N.E., Pérez-Carrillo E., Marín-Obispo L.M., Hernández-Brenes C., Santacruz A., Jacobo-Velázquez D.A. (2021). Sugar-Free Milk Chocolate as a Carrier of Omega-3 Polyunsaturated Fatty Acids and Probiotics: A Potential Functional Food for the Diabetic Population. Foods.

[13] Mlcek J., Plaskova A., Jurikova T., Sochor J., Baron M., Ercisli S. (2021). Chemical, Nutritional and Sensory Characteristics of Six Ornamental Edible Flowers Species. Foods.

[14] Baraniak J., Kania-Dobrowolska M. (2022). The Dual Nature of Amaranth—Functional Food and Potential Medicine. Foods.

